# Fungal Biovalorization of a Brewing Industry Byproduct, Brewer’s Spent Grain: A Review

**DOI:** 10.3390/foods10092159

**Published:** 2021-09-13

**Authors:** Andrew Marcus, Glen Fox

**Affiliations:** Food Science and Technology Department, University of California, One Shields Ave, Davis, CA 95616, USA; anmarcus@ucdavis.edu

**Keywords:** brewer’s spent grain, brewing, fungal biovalorization, food waste, malt

## Abstract

The beer industry is a major producer of solid waste globally, primarily in the form of brewer’s spent grain (BSG), which due to its low value has historically been diverted to livestock as feed or to landfills. However, its high moisture content and chemical composition positions BSG as an ideal candidate for further processing with microbial fermentation. Recent research has focused on filamentous fungi and the ability of some species therein to degrade the predominant recalcitrant cellulolignin components of BSG to produce valuable compounds. Many species have been investigated to biovalorize this waste stream, including those in the genuses *Aspergillus*, *Penicillium*, *Rhyzopus,* and *Trichoderma*, which have been used to produce a wide array of highly valuable enzymes and other functional compounds, and to increase the nutritional value of BSG as an animal feed. This review of recent developments in the application of filamentous fungi for the valorization of BSG discusses the biochemical makeup of BSG, the biological mechanisms underlying fungi’s primacy to this application, and the current applications of fungi in this realm.

## 1. Introduction

**Figure 1:**
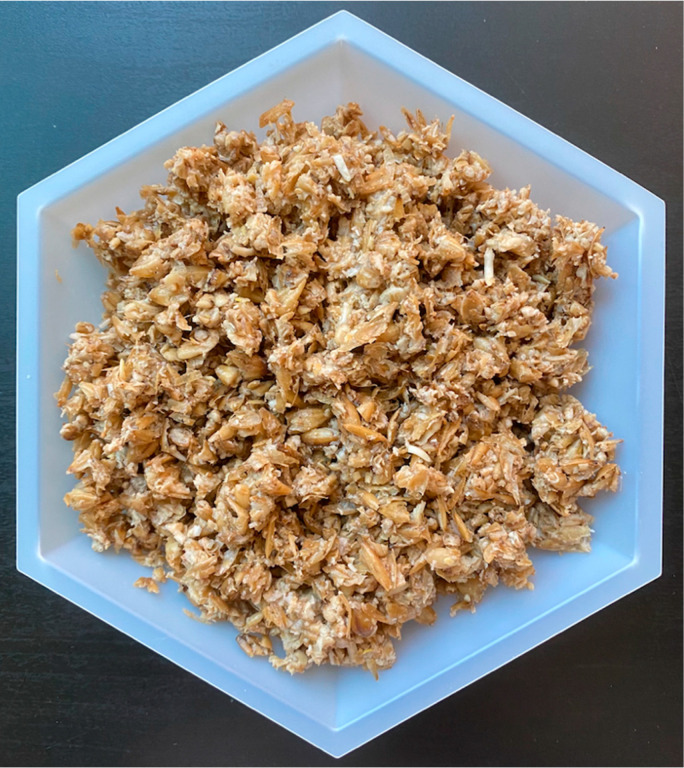
Brewer’s spent grain (BSG) consisting of barley malt removed from the lauter tun at the UC Davis Pilot Brewery. Photo courtesy of Emily Newman. Written permission was granted to the authors.

Though the process of brewing beer has been present in human culture for many generations, the composition of its byproducts has not changed significantly. Beer is produced using a combination of enzymatic and microbial fermentation processes to transform raw grains into a final product rich in carbohydrates, mainly in the form of oligosaccharides, and ethanol. Prior to brewing, these grains—mainly barley—are malted, where a process of partial germination begins to break down storage structures and enzymes are synthesized. At the brewery, this malt is milled and added to warm water to create a slurry called the mash, where the endogenous enzymes convert starches into fermentable sugars, mostly consisting of maltose and maltotriose, and non-fermentable oligosaccharides, colloquially referred to as dextrins. Additionally, proteins are partially digested to peptides and amino acids. Following this step, the liquid portion, known as wort, is drained, separated from the insoluble materials, boiled, and finally fermented to produce ethanol and other byproducts before being packaged as beer [1]. The solid fraction remaining after the extraction of the wort consists primarily of barley husks and other insoluble materials [1], and is known as brewer’s spent grain (BSG) (Figure 1). The processing of grains in a brewery is primarily focused on the extraction of the products of hydrolyzed starch, with some care given to other minor compounds, such as soluble proteins and free amino acids. However, the solid waste stream produced by this industry is concentrated in other functional compounds not relevant to beer production.

The global beer industry is reported to produce over 2.3 × 10^11^ L of beer annually [2]. BSG represents an estimated 85% of the solid waste produced from brewery operations, while the other 15% is represented by trub (precipitated protein and insoluble materials after boiling), spent hops, spent yeast, and adsorbent solids from filtration, including diatomaceous earth [3]. BSG is generated from beer at an estimated rate of 19.7% by weight (kg BSG per kg beer produced) [4], resulting in an estimated generation of 4.5 × 10^10^ kg of BSG annually worldwide.

Once the mash has had all the available wort extracted, the remaining BSG has historically been of low commercial value and typically has been disposed of as waste in landfills, incurring significant costs to the brewery, or has been sold or given away as animal feed [5]. Neither of these processes represent the most efficient use of this byproduct and may actually produce further negative environmental impacts. As animal feed, it is primarily destined for ruminants, such as cattle, which are known to be significant producers of greenhouse gasses, and as a group are identified as the single largest anthropogenic source of methane [6]. Ruminant livestock alone in the US are estimated to produce 28% of the total methane emitted annually [7]. Further research is required to compare the methane produced by ruminants fed BSG compared to other feeds, though it is potentially similar. If BSG is diverted to landfill, the addition of this highly fermentable waste increases the already unmanaged anaerobic fermentation there, where greenhouse gases, such as carbon dioxide and methane, are emitted [8]. Other potential avenues have been proposed for the re-routing of BSG as a byproduct, including those reviewed by Mussatto et al., [5], such as including building materials [9], charcoal [5], material for paper manufacture [10], and energy generation through direct combustion [11], and others as reviewed by Jackowski et al., [12], including hydrothermal carbonization for energy generation and soil amendments.

However, none of these have yet been implemented widely, primarily due to the high moisture content of BSG as it exits the brewery. At 75–80% dry basis moisture content [13] and as drying can be costly [14], it has not been considered appropriate for combustion-based energy generation [11]. Coupled with the dispersed geographical nature of breweries, substantial financial and energetic costs would be incurred in transportation to a disposal or processing facility. In addition to the high moisture content, a lack of cooling of BSG as it leaves the brewery risks microbial spoilage during storage and transfer [15]. BSG held at room temperature (20 °C) has been shown to exhibit extensive microbial activity [16]. 

Controlled microbial fermentation, however, is a promising strategy to valorize this high-volume waste stream. Already, when transferred to landfill or animal feed, the power of microbial fermentation is being leveraged to transform the substrate. In landfill, microbes reduce the BSG volume, and as animal feed, rumen microbiota act to degrade BSG into more bioavailable nutrients for the animal [17]. It is, however, through the unmanaged nature of these processes that the maximum potential commercial value of BSG is not realized, nor are the negative externalities mitigated. By employing specific fungal species in controlled settings, BSG is an ideal substrate to produce a wide variety of high-value products.

## 2. Composition of BSG

BSG is produced year-round and globally, though its chemical composition can be highly variable due to many factors. Physically, BSG is made up of the largely recalcitrant barley husk, but also portions of the grain endosperm, which contains small amounts of starch, proteins and lipids [5]. As shown in Table 1, BSG is mainly composed of non-starch polysaccharides (NSPs), including cellulose, beta-glucan, and hemicellulose, primarily represented by arabinoxylan. Combined, these compounds may contribute to over 60% of the dry weight of BSG [13]. Arabinoxylan is the most abundant NSP, at 21–30%, with lignin second at 12–22% [13]. Importantly, the protein content of BSG is also prominent, at 10–26% [13]. The large variability in all components reflects the heterogeneity of BSG; it can vary based on the barley variety, harvest time, growing conditions, addition of other grains or starches added to the mash, and the malting and mashing conditions [13].

Cellulose, hemicellulose and lignin make up most of the cell walls of plants and act to maintain structural rigidity. They are collectively referred to as lignocellulosic biomass. Cellulose is the simplest of these polymers and consists of chains of glucose with β(1→4) glycosidic linkages up to hundreds of glucose monomers long [26]. Hemicellulose is a branched heteropolymer that is primarily represented by arabinoxylan in BSG. Arabinoxylan is composed of a backbone of β(1→4)-D-xylose, which can be di-or mono-substituted with α-L-arabinose. The polymer is highly heterogeneous, and may also have many substituents, including uronic acids, phenolic acids, acetyl groups or proteins [27]. Lignin is a phenolic polymer of greater complexity than hemicellulose and is made up of phenylpropanoid units, such as *p*-coumaryl, coniferyl, and sinapyl alcohol. Beyond its structural rigidity, lignin is specifically resistant to microbial attack through non-specific adsorption and binding of hydrolytic enzymes and through the toxicity of lignin derivatives [28]. Cellulose is frequently embedded into the lignocellulosic matrix, which increases its resistance to enzymatic hydrolysis [28]. Furthermore, because both hemicellulose and lignin are heterogeneous in their branching and may have huge ranges of degrees of polymerization, they are generally resistant to enzymatic degradation [29]. 

Additionally, the biochemical makeup of BSG is not unique to other agricultural and industrial waste streams, and thus similar fungal biovalorization techniques might be applied to a number of other substrates. Other grain processing industries, for example, produce byproducts of similar lignocellulosic makeup, including wet corn distillers grain, a byproduct of ethanol production [30]. Furthermore, the remaining agricultural residue after grains are harvested, such as barley straw, is high in these lignocellulosic compounds [29]. An assortment of other agricultural byproducts and their cell wall components are listed in Table 2. 

## 3. Roles for Fungal Transformation of BSG

Plant polymers, such as cellulose, hemicellulose, and lignin, though indigestible to humans, have long been used as nutrient sources for many microorganisms [39]. Fungi, in particular, have been specifically targeted for use with BSG as a substrate, as they are well known to digest similar compounds in their natural environment [39]. The heteropolymeric nature of both hemicellulose and lignin requires a battery of enzymes, each with a specific activity to hydrolyze the different types of bonds [29]. Arabinoxylan is considered difficult to degrade by microorganisms due to, for example, its structural complexity, yet filamentous fungi have been shown to produce an array of enzymes that work synergistically to fully degrade the polymer. Xylanase enzymes in fungi have been reviewed by Knob et al., 2010 [40], the most relevant of which are endo-β-1,4-xylanase and β-D-xylosidase, but also include accessory enzymes, such as α-L-arabinofuranosidase and β-glucuronidase, that act to cleave subunits off the main xylan chain.

For this reason, filamentous fungi are considered prime candidates for the degradation of lignocellulosic-rich BSG substrate and provide a more cost- and energy-efficient alternative to systems that apply a combination of heat, chemicals and purchased enzymes [41]. The most cost-effective bioreactor model is solid-state fermentation (SSF), where the media contain little to no free-flowing liquid between the solid particles, and the solid density is very high [42]. This is particularly well-suited for filamentous fungi [31], as their hyphae can penetrate the spaces between particles [43]. Additionally, the water activity and moisture content of untreated BSG leaving the brewery is within the range of typical SSF media [42], and would not require additional drying or water addition.

Research into the use of fungi with BSG as a substrate for industrial purposes may be classified into three different categories for the purpose of this review: (1) the production of enzymes; (2) the production of other valuable compounds, including bioethanol; and (3) to increase the nutritional quality of the BSG for animal feed (Table 3). Some of these processes can even achieve several goals simultaneously, and different species of microbes may be used in tandem to that end. 

## 4. Enzyme Production

The production of enzymes has been proven to be the most interesting and well-researched potential products of BSG fermentation, as enzymes have a myriad of applications in food, pharmaceutical, and chemical industries. A challenge for the cost-effectiveness of these industrial processes is the large amounts of enzymes that are required. For example, an estimated 25% of the cost of second generation (lignocellulosic) bioethanol production is solely from the purchase of enzymes used to degrade the lignocellulosic material [67]. Filamentous fungi are particularly well-suited to the production of enzymes for later extraction for a few reasons. First, many of their plant cell wall-degrading enzymes (Table 2) are secreted extracellularly into the substrate medium, which allows for their extraction without the need to disrupt the fungal cells downstream [40]. Second, to a greater degree than yeasts and bacteria, fungi are known to produce extracellular enzymes at much higher concentrations [40], and thus their yield can be much greater.

Fungi have been used to produce enzymes industrially for many years [39], though the use of fungi with BSG for enzyme production is a newer application. Species across many genuses (Table 3), including *Aspergillus*, *Penicillium* and *Fusarium,* have been investigated separately for enzyme production at lab scale with BSG substrate in recent decades with positive results [13]. The most frequently investigated enzymes are those used to break down lignocellulosic material. *Aspergillus flavus* has been used in SSF bioreactors with BSG to produce cellulase enzymes [47], and both *Moesziomyces antarcticus* and Moesziomyces aphidis have been shown to produce xylanases at very high activity when using BSG as a substrate [57]. Interestingly, ligninolytic enzymes are known to be produced by many species of fungi [68], yet little research has been conducted on BSG as a substrate for its production and may indicate a potential avenue for future research.

The value of these fungi-derived enzymes produced with BSG requires further research if they are to be used for industrial purposes. Generally, many enzymes exhibit enhanced activity at higher temperatures, and those higher temperatures can also inhibit unwanted microbial growth [29]. Many xylanolytic enzymes produced by fungi on different media are, however, not heat stable [39]. In general, the majority of microbial enzymes used in industrial processes are mesophilic, and used from 35–60 ℃ [69]. Additionally, many of these studies have only proven the viability of lab-scale processes, and the scale-up of many of them will prove challenging. The scale-up of BSG fermentation tanks can lead to increased oxygen transfer into the substrate and, therefore, increased xylanase production for some bacterial species [70], but it is not known how this will affect filamentous fungi.

## 5. Bioethanol and Other Products

BSG can also be used by fungi for bioethanol production. Some species of fungi have been investigated for their ability to produce enzymes, as well as to degrade the lignocellulosic material. After BSG polymers have been broken down into smaller constituent parts, some fungi are able to further extract energy through the fermentation of those remaining BSG parts into ethanol. *Neurospora crassa*, a mold of the phylum Ascomycota has been known for some time to have the ability to simultaneously convert lignocellulosic material into fermentable sugars, and then ferment those sugars into ethanol [59]. More recently, *N. crassa* has been proven to be applicable in BSG fermentation, with an optimized method utilizing enzyme production in SSF bioreactors, followed by lignocellulose hydrolysis and ethanol production in a submerged-state bioreactor [24].

In other studies, it has been proposed that a combined approach may be applied to produce multiple product streams, which incorporate not only fungal processes, but chemical hydrolysis and bacterial fermentations in tandem or in sequence. Dávila et al., [41] developed a model in which a theoretical BSG plant could incorporate the breakdown of both arabinoxylan and cellulose into the highly valuable products xylitol, ethanol and polyhydroxybuterate. In this study, the BSG was chemically pre-treated with an acid to produce a xylose-rich hydrosylate from arabinoxylan that was then fermented by *Candida guilliermondii* yeasts to produce xylitol. This yeast is an ideal candidate for the xylitol production step, as it produces xylose reductase (EC.1.1.1.21), which is well known to produce high yields of xylitol from xylose from a multitude of sources [71]. In this proposed model, the cellulose fraction is physically separated and processed with the mold *Trichoderma reesei* to produce glucose. The glucose is then routed to two separate bioreactors to produce different products from the glucose. In one of the bioreactors, the yeast *Zymomonas mobilis* ferments approximately 60% of the glucose to produce ethanol, and the remaining glucose is processed into polyhodroxybuterate by the bacterium *Cupriavidus necatur*.

Species in the fungal genus *Tricoderma*, including *Trichoderma reesei*, have been used for decades for bioethanol production [72], but only with their purified cellulase enzymes produced industrially. However, to date, they have not been used as extensively in ethanol-producing bioreactors to break down biomass such as BSG. Furthermore, the genome sequencing of this species has shown that up to ten genes for different cellulases and hemicellulases are present in the genome, though only four of them are produced at sufficient quantities for industrial production [73]. The presence of similar genes that produce these enzymes has also been investigated for cellulase production in other fungal species. This includes genes from *Aspergillus foetidus*, and a number of *Penicillium* species, including *P. verruculosum*, *P. pinophilum*, *P. funiculosum* and *P. echinulatum*, with varying success [73].

## 6. Partial BSG Degradation for Enhanced Feed Quality

Currently, the most likely disposal route for BSG utilization is as animal feed, particularly for cattle. It has also been proposed for use with pigs, poultry and even fish [74]. However, the value of BSG may increase through the bioconversion of some compounds with fungi. Feed processing methods have been developed that use feed substrates that are partially hydrolyzed by, and still contain, active cellulase and hemicellulose enzymes but no live fungi. The addition of these enzymes to feed for dairy cattle biochemically similar to BSG has been shown to increase milk yield, presumably by increasing the digestibility as the enzymes remain active in the gastrointestinal tract [31,75]. Similarly, the pre-treatment of animal feed by fungal fermentation can increase the quality of the feed itself, especially in protein content, but also in other micronutrients. The *Aspergillus* species *A. oryzae* and *A. awamori* have been shown to increase the protein content of BSG by 20–36% as a result of increased fungal biomass [66]. Other studies have shown that *Rhizopus oligosporus* is also capable of increasing both crude protein and soluble protein by approximately two times that of the original BSG for similar reasons [76]. More recently, fermentation using *R. oligosporus* has been shown to further enhance nutritional value by increasing the concentration of some amino acids, citric acid, vitamins, and antioxidants [65].

## 7. Conclusions

BSG is a significant byproduct of the brewing industry that has historically been rele-gated to the cheapest forms of food waste disposal. Due to its high moisture and lig-nocellulosic content, it is most frequently diverted as low quality animal feed or simply to landfill. However, through the use of filamentous fungi, this substrate has the potential to yield many high value products, or even increase its value as feed through bioconversion. A wide range of species have been studied for their ability to use BSG as a substrate in-cluding those from the genera Aspergillus, Trichoderma, Neurospora, Candida, and Rhizopus, among others. These species have been used to produce a diversity of recoverable enzymes from BSG, including α- and β-amylases, cellulases, hemicellulases, proteases and xy-lanases. Other functional compounds such as glucose, xylose, xylitol, and citric acid have also been recovered from BSG treated with these fungi, and some of these compounds have been later used to produce bioethanol. Additionally, fungi may be leveraged for their ability to increase the nutrient quality of BSG for livestock feed through the digestion of hemicellulose and the production of proteins, amino acids, antioxidants, and vitamins. Though the techniques developed for these processes are specific to the composition of BSG, it is the functionality of the fungal species used that indicates the potential for ex-pansion of these techniques to similar and abundant waste streams also associated with the brewing industry and high in lignocellulosic materials, such as the agricultural resi-dues from barley, corn, rice, and other cereals. With further research into this field, as well as addressing the challenges to scaling-up and transportation of BSG from breweries, this process may be applied more widely, and beer production may become more environmen-tally sustainable. 

## Figures and Tables

**Table 1:** Variability in composition of brewer’s spent grain. All values are expressed in g per 100 g dry matter (% *w*/*w*). Table adapted from Xiros & Christakopoulos [13] using data reported by Kanauchi et al., 2001 [18], Santos et al., 2003 [19], Carvalheiro et al., 2004 [20], Mussatto & Roberto 2005 [21], Celus et al., 2006 [22], Jay et al., 2008 [23], Xiros et al., 2008 [24], and Robertson et al., 2010 [25].

Component	Lowest Published Value	Highest Published Value
protein	10.0	26.7
lipids	3.0	10.6
starch	1.0	13.0
ash	1.2	4.6
non-starch glucans (incl. cellulose)	0.3	21.9
arabinoxylan (hemicellulose)	21.0	29.6
lignin	11.9	22.0
phenolics	0.7	2.0

**Table 2:** Crop residues from plant species used for beer brewing and their cell wall components. Many of these contain cellulose, hemicellulose and lignin in concentrations similar to BSG. All values are expressed in g per 100 g dry matter (% *w*/*w*). Table adapted from Graminha et al., 2008 [31] using data reported by Cruz 1992 [32], Prates 1995 [33], Cruz et al., 2000 [34], Couto & Sanromán 2005 [35], Reddy & Yang 2005 [36], Karimi et al., 2006 [37], and Tabka et al., 2006 [38].

Crop	Residue Fraction	Cellulose	Hemicellulose	Lignin
Barley	straw	31.0–45.0	25.4–38.0	11.0–19.0
	bran	23.0	32.0	21.4
Wheat	straw	27.0–40.0	21.0–32.0	9.8–20.0
	bran	30.0	50.0	15.0
Rice	straw	28.0–47.0	19.0–28.0	4.3–24.0
	bran	35.0	25.0	17.0
Corn	straw	33.5	24.9	7.8
	bran	33.8	39.3	4.9
	silage	38.0–40.0	28.0	7.0–21.0
	stalk	33.6	23.7	8.7
	leaf	24.5	27.3	5.4
	cob	37.7	39.6	7.3
Oats	straw	30.0	22.0	8.5
	bran	49.3	25.0	18.0
Sorghum	stalk	27.0	25.0	11.0

**Table 3:** Fungal species and the end products of fermentation of BSG, as separated into different production categories.

Production Category	End Product	Species Used	Reference
enzymes	α-amylase	*Aspergillus oryzae*	Xu et al., 2008 [44] Patel et al., 2005 [45]
	amyloglucosidase	*Aspergillus fumigatus*	Adeniran et al., 2010 [46]
	β-amylase	*Aspergillus niger*	Orji et al., 2016 [47]
		*Helminthosporium oxysporium*	
		*Penicillium frequestans*	
		*Aspergillus fumigatus*	
	cellulases	*Aspergillus niger*	
		*Helminthosporium oxysporium*	
		*Penicillium frequestans*	
		*Aspergillus flavus*	
	glucanases	*Aspergillus fumigatus*	Grigorevski-Lima et al., 2009 [48]
		*Fusarium oxysporum*	Xiros et al., 2008 [44]
		*Neurospora crassa*	Benko et al., 2007 [49]
		*Trichoderma reesei*	
		*Trichoderma* spp	Napolitano et al., 2006 [50]
	hemicellulases	*Fusarium oxysporum*	Xiros and Christakopoulos 2009 [51]
		*Neurospora crassa*	Xiros et al., 2008 [44]
		*Penicillium brasilianum*	Panagiotou et al., 2006 [52]
		*Penicillium janczewskii*	Terrasan et al., 2010 [53]
		*Talaromyces stipitatus*	Mandalari et al., 2005 [54]
		*Trichoderma* spp	Napolitano et al., 2006 [50]
	laccases	*Trametes versicolor*	Tišma et al., 2018 [55]
	proteases	*Aspergillus oryzae*	Sandhya et al., 2005 [56]
	xylanases	*Moesziomyces antarcticus*	Faria et al., 2019 [57]
		*Moesziomyces aphidis*	
		*Penicillium janczewskii*	Terrasan and Carmona 2015 [58]
other products	bioethanol	*Neurospora crassa*	Deshpande et al., 1986 [59], Xiros et al., 2008 [44]
	ethanol (for bioethanol)	*Zymomonas mobilis*	Dávila et al., 2016 [41]
	glucose (for bioethanol)	*Trichoderma reesei*	
	xylose (for bioethanol)	*Candida guilliermondii*	
	citric acid	*Aspergillus niger*	Dhillon et al., 2011 [60], Pathania et al., 2018 [61]
	single cell protein	*Aspergillus niger*	Aregbesola and Omafuvbe 2014 [62]
	xylitol	*Candida guilliermondii*	Mussatto and Roberto 2005 [21]
		*Trichoderma reesei*	Amorim et al., 2019 [63]
		*Komagataella pastoris*	Araújo et al., 2021 [64]
nutrient-enhanced feed	hemicellulose digestion	*Penicillium janczewskii*	Terrasan and Carmona 2015 [58]
	increased amino acids citric acid antioxidants and vitamins	*Rhizopus oligosporus*	Cooray and Chen, 2018 [65]
	increased protein content	*Aspergillus awamori*	Bekatorou et al., 2007 [66]
		*Aspergillus oryzae*	

## References

[1] Bamforth C. (2006). Scientific Principles of Malting & Brewing.

[2] FAO FAOSTAT Crops Processed FAOSTAT. http://www.fao.org/faostat/en/#home.

[3] Lynch K.M., Steffen E.J., Arendt E.K. (2016). Brewers’ spent grain: A review with an emphasis on food and health. J. Inst. Brew..

[4] Niemi P., Faulds C.B., Sibakov J., Holopainen U., Poutanen K., Buchert J. (2012). Effect of a milling pre-treatment on the enzymatic hydrolysis of carbohydrates in brewer’s spent grain. Bioresour. Technol..

[5] Mussatto S.I., Dragone G., Roberto I.C. (2006). Brewers’ spent grain: Generation, characteristics and potential applications. J. Cereal Sci..

[6] Mathison G.W., Okine E.K., McAllister T.A., Dong Y., Galbraith J., Dmytruk O.I. (1998). Reducing Methane Emissions from Ruminant Animals. J. Appl. Anim. Res..

[7] Overview of Greenhouse Gases | Greenhouse Gas (GHG) Emissions | US EPA. https://www.epa.gov/ghgemissions/overview-greenhouse-gases#methane.

[8] Lee U., Han J., Wang M. (2017). Evaluation of landfill gas emissions from municipal solid waste landfills for the life-cycle analysis of waste-to-energy pathways. J. Clean. Prod..

[9] Russ W., Mörtel H., Meyer-Pittroff R. (2005). Application of spent grains to increase porosity in bricks. Constr. Build. Mater..

[10] Ishiwaki N., Murayama H., Awayama H., Kanauchi O., Sato T. (2000). Development of high value uses of spent grain by fractionation technology. Tech. Q.-Master Brew. Assoc. Am..

[11] Zanker G., Kepplinger W., Pecher C., Oreopoulou V., Russ W. (2007). Incineration of solid food waste: A project about spent grain. Utilization of By-Products and Treatment of Waste in the Food Industry.

[12] Jackowski M., Niedźwiecki Ł., Jagiełło K., Uchańska O., Trusek A. (2020). Brewer’s Spent Grains-Valuable Beer Industry By-Product. Biomolecules.

[13] Xiros C., Christakopoulos P. (2012). Biotechnological potential of brewers spent grain and its recent applications. Waste Biomass Valor..

[14] Aboltins A., Palabinskis J. (2015). Research in brewer’s spent grain drying process. Eng. Rural. Dev..

[15] Sodhi H.S., Garcha H.S., Kiran U. (1985). Screening of mycoflora of spent-up brewers’ grains for aflatoxin production. J. Res. Punjab Agric. Univ..

[16] Robertson J.A., I’Anson KJ A., Brocklehurst T.F., Faulds C.B., Waldron K.W. (2010). Effect of storage conditions on the microbial ecology and biochemical stability of cell wall components in brewers’ spent grain. J. Agric. Food Chem..

[17] Iqbal M.F., Cheng Y.-F., Zhu W.-Y., Zeshan B. (2008). Mitigation of ruminant methane production: Current strategies, constraints and future options. World J. Microbiol. Biotechnol..

[18] Kanauchi O., Mitsuyama K., Araki Y. (2001). Development of a Functional Germinated Barley Foodstuff from Brewer’s Spent Grain for the Treatment of Ulcerative Colitis. ASBCJ..

[19] Santos M., Jiménez J. J., Bartolomé B., Gómez-Cordovés C., del Nozal M. J. (2003). ; Variability of brewer’s spent grain within a brewery. Food Chem..

[20] Carvalheiro F., Esteves M.P., Parajó J.C., Pereira H., Gírio F.M. (2004). Production of oligosaccharides by autohydrolysis of brewery’s spent grain. Bioresour. Technol..

[21] Mussatto S.I., Roberto I.C. (2005). Acid hydrolysis and fermentation of brewer’s spent grain to produce xylitol. J. Sci. Food Agric..

[22] Celus I., Brijs K., Delcour J. A. (2006). The effects of malting and mashing on barley protein extractability. J Cereal Sci.

[23] Jay A.J., Parker M.L., Faulks R., Husband F., Wilde P., Smith A.C., Faulds C.B., Waldron K.W. (2008). A systematic micro-dissection of brewers’ spent grain. J Cereal Sci..

[24] Xiros C., Topakas E., Katapodis P., Christakopoulos P. (2008). Hydrolysis and fermentation of brewer’s spent grain by Neurospora crassa. Bioresour. Technol..

[25] Robertson J. A., I’Anson K. J. A., Treimo J., Faulds C. B., Brocklehurst T. F., Eijsink V. G. H., Waldron K. W. (2010). Profiling brewers’ spent grain for composition and microbial ecology at the site of production. LWT..

[26] You C., Chen H., Myung S., Sathitsuksanoh N., Ma H., Zhang X.-Z., Li J., Zhang Y.-H.P. (2013). Enzymatic transformation of nonfood biomass to starch. Proc. Natl. Acad. Sci. USA.

[27] Fincher G.B., Stone B.A. (1986). Cell walls and their components in cereal grain technology. Adv. Cereal Sci. Technol..

[28] Zoghlami A., Paës G. (2019). Lignocellulosic biomass: Understanding recalcitrance and predicting hydrolysis. Front. Chem..

[29] Beg Q.K., Kapoor M., Mahajan L., Hoondal G.S. (2001). Microbial xylanases and their industrial applications: A review. Appl. Microbiol. Biotechnol..

[30] Flodman H.R., Noureddini H. (2013). Effects of intermittent mechanical mixing on solid-state fermentation of wet corn distillers grain with Trichoderma reesei. Biochem. Eng. J..

[31] Graminha E., Gonçalves A., Pirota R., Balsalobre M., Da Silva R., Gomes E. (2008). Enzyme production by solid-state fermentation: Application to animal nutrition. Anim. Feed Sci. Technol..

[32] da Cruz G.M. (1992). Utilização de restos de culturas e palhas na alimentação de ruminantes. Proceedings of the Embrapa Pecuária Sudeste-Artigo em anais de congresso (ALICE).

[33] Prates E.R. (1995). Arroz e cereais de inverno. Simpósio sobre nutrição de Bovinos.

[34] Cruz J. M., Domínguez J. M., Domínguez H., Parajó J. C. (2000). Preparation of fermentation media from agricultural wastes and their bioconversion into xylitol. Food Biotechnol..

[35] Rodríguez Couto S., Sanromán M. A. (2005). Application of solid-state fermentation to ligninolytic enzyme production. Biochem Eng J..

[36] Reddy N., Yang Y. (2005). Biofibers from agricultural byproducts for industrial applications. Trends Biotechnol..

[37] Karimi K., Kheradmandinia S., Taherzadeh M. J. (2006). Conversion of rice straw to sugars by dilute-acid hydrolysis. Biomass Bioenergy.

[38] Tabka M.G., Herpoël-Gimbert I., Monod F., Asther M., Sigoillot J.C. (2006). Enzymatic saccharification of wheat straw for bioethanol production by a combined cellulase xylanase and feruloyl esterase treatment. Enzyme Microb. Technol..

[39] Polizeli M. L. T. M., Rizzatti A. C. S., Monti R., Terenzi H. F., Jorge J. A., Amorim D. S. (2005). Xylanases from fungi: properties and industrial applications. Appl. Microbiol. Biotechnol..

[40] Knob A., Terrasan C. R. F., Carmona E. C. (2010). β-Xylosidases from filamentous fungi: an overview. World J Microbiol Biotechnol..

[41] Dávila J.A., Rosenberg M., Cardona C.A. (2016). A biorefinery approach for the production of xylitol, ethanol and polyhydroxybutyrate from brewer’s spent grain. AIMS Agric. Food.

[42] Lonsane B.K., Ghildyal N.P., Budiatman S., Ramakrishna S.V. (1985). Engineering aspects of solid state fermentation. Enzym. Microb. Technol..

[43] Muller dos Santos M., Souza da Rosa A., Dal’Boit S., Mitchell D.A., Krieger N. (2004). Thermal denaturation: Is solid-state fermentation really a good technology for the production of enzymes?. Bioresour. Technol..

[44] Xu Z.M., Emmanouelidou D.G., Raphaelides S.N., Antoniou K.D. (2008). Effects of heating temperature and fat content on the structure development of set yogurt. J. Food Eng..

[45] Patel A.K., Nampoothiri K.M., Ramachandran S., Szakacs G., Pandey A. (2005). Partial purification and characterization of-amylase produced by Aspergillus oryzae using spent-brewing grains. Indian J. Biotechnol..

[46] Adeniran H.A., Abiose S.H., Ogunsua A.O. (2010). Production of Fungal β-amylase and Amyloglucosidase on Some Nigerian Agricultural Residues. Food Bioprocess Technol..

[47] Orji F., Dike E., Lawal A., Sadiq A., Fashola F., Suberu Y., Famotemi A., Ita B., Ugbana A., Adefiranye A. (2016). Properties of Aspergillus flavus Cellulase Produced from Solid State Fermentation of Brewers’ Spent Grain (BSG) as Substrate. Br. Biotechnol. J..

[48] Grigorevski-Lima A.L., Da Vinha F.N.M., Souza D.T., Bispo A.S.R., Bon E., Coelho R.R.R., Nascimento R.P. (2009). Aspergillus fumigatus thermophilic and acidophilic endoglucanases. Appl. Biochem. Biotechnol..

[49] Benkő Z., Drahos E., Szengyel Z., Puranen T., Vehmaanperä J., Réczey K., Mielenz J.R., Klasson K.T., Adney W.S., McMillan J.D. (2007). Thermoascus aurantiacus CBHI/Cel7A Production in Trichoderma reesei on Alternative Carbon Sources. Applied biochemistry and Biotecnology.

[50] Napolitano A., Lanzuise S., Ruocco M., Arlotti G., Ranieri R., Knutsen S. H., Lorito M., Fogliano V. (2006). Treatment of cereal products with a tailored preparation of trichoderma enzymes increases the amount of soluble dietary fiber. J. Agric. Food Chem..

[51] Xiros C., Christakopoulos P. (2009). Enhanced ethanol production from brewer’s spent grain by a Fusarium oxysporum consolidated system. Biotechnol. Biofuels.

[52] Panagiotou G., Granouillet P., Olsson L. (2006). Production and partial characterization of arabinoxylan-degrading enzymes by Penicillium brasilianum under solid-state fermentation. Appl. Microbiol. Biotechnol..

[53] Terrasan C.R.F., Temer B., Duarte M.C.T., Carmona E.C. (2010). Production of xylanolytic enzymes by Penicillium janczewskii. Bioresour. Technol..

[54] Mandalari G., Faulds C.B., Sancho A.I., Saija A., Bisignano G., LoCurto R., Waldron K.W. (2005). Fractionation and characterisation of arabinoxylans from brewers’ spent grain and wheat bran. J. Cereal Sci..

[55] Tišma M., Jurić A., Bucić-Kojić A., Panjičko M., Planinić M. (2018). Biovalorization of brewers’ spent grain for the production of laccase and polyphenols. J. Inst. Brew..

[56] Sandhya C., Sumantha A., Szakacs G., Pandey A. (2005). Comparative evaluation of neutral protease production by Aspergillus oryzae in submerged and solid-state fermentation. Process. Biochem..

[57] Faria N.T., Marques S., Ferreira F.C., Fonseca C. (2019). Production of xylanolytic enzymes by Moesziomyces spp. using xylose, xylan and brewery’s spent grain as substrates. New Biotechnol..

[58] Terrasan C.R.F., Carmona E.C. (2015). Solid-state fermentation of brewer’s spent grain for xylanolytic enzymes production by Penicillium janczewskii and analyses of the fermented substrate. Biosci. J..

[59] Deshpande V., Keskar S., Mishra C., Rao M. (1986). Direct conversion of cellulose/hemicellulose to ethanol by Neurospora crassa. Enzym. Microb. Technol..

[60] Dhillon G.S., Brar S.K., Verma M., Tyagi R.D. (2011). Utilization of different agro-industrial wastes for sustainable bioproduction of citric acid by Aspergillus niger. Biochem. Eng. J..

[61] Pathania S., Sharma S., Kumari K. (2018). Solid state fermentation of BSG for citric acid production. Indian J. Nat. Prod. Resour. (IJNPR) [Former. Nat. Prod. Radiance (NPR)].

[62] Aregbesola O.A., Omafuvbe B.O. (2014). Production of Aspergillus niger biomass from aqueous extract of brewer’s spent grain. Ife J. Sci..

[63] Amorim C., Silvério S.C., Rodrigues L.R. (2019). One-step process for producing prebiotic arabino-xylooligosaccharides from brewer’s spent grain employing Trichoderma species. Food Chem..

[64] Araújo D., Costa T., Freitas F. (2021). Biovalorization of Lignocellulosic Materials for Xylitol Production by the Yeast Komagataella pastoris. Appl. Sci..

[65] Cooray S.T., Chen W.N. (2018). Valorization of brewer’s spent grain using fungi solid-state fermentation to enhance nutritional value. J. Funct. Foods.

[66] Bekatorou A., Bountas Y., Banat I., Kanellakl M. (2007). Upgrading brewer’s spent grains by treatment with Aspergillus species. Chem. Ind. Chem. Eng. Q./CICEQ.

[67] Humbird D., Davis R., Tao L., Kinchin C., Hsu D., Aden A., Schoen P., Lukas J., Olthof B., Worley M. (2011). Process Design and Economics for Biochemical Conversion of Lignocellulosic Biomass to Ethanol: Dilute-Acid Pretreatment and Enzymatic Hydrolysis of Corn Stover.

[68] Górska E.B., Jankiewicz U., Dobrzyński J., Gałązka A., Sitarek M., Gozdowski D., Russel S., Kowalczyk P. (2014). Production of ligninolytic enzymes by cultures of white rot fungi. Pol. J. Microbiol..

[69] Walsh G. (2015). Proteins: Biochemistry and Biotechnology.

[70] Moteshafi H., Mousavi S.M., Hashemi M. (2019). Aeration challenge in high BSG suspended fermentation: Impact of stirred-tank bioreactor scale. Biomass Bioenergy.

[71] Rosa SM A., Felipe MG A., Silva S.S., Vitolo M. (1998). Xylose reductase production by candida guilliermondii. Appl. Biochem. Biotechnol..

[72] Mandels M., Sternberg D. (1976). Recent advances in cellulase technology. J. Ferment. Technol..

[73] Gusakov A.V. (2011). Alternatives to Trichoderma reesei in biofuel production. Trends Biotechnol..

[74] Kaur V. (2004). Incorporation of brewery waste in supplementary feed and its impact on growth in some carps. Bioresour. Technol..

[75] Rode L.M., Yang W.Z., Beauchemin K.A. (1999). Fibrolytic enzyme supplements for dairy cows in early lactation. J. Dairy Sci..

[76] Canedo M.S., de Paula F.G., da Silva F.A., Vendruscolo F. (2016). Protein enrichment of brewery spent grain from Rhizopus oligosporus by solid-state fermentation. Bioprocess Biosyst. Eng..

